# Supplementary material for: Splice-Junction-Based Mapping of Alternative Isoforms in the Human Proteome
Source: Cell Rep. Author manuscript; Available in PMC 2020 Jan 15. (PMC6961840; doi:10.1016/j.celrep.2019.11.026)
Supplement: 2 [file NIHMS1546469-supplement-2.zip › DF1/PXD009737/TestisTrypsin/TestisTrypsin_38_THBS1_DLSLATSPAAAFMLLFLMHVCGTNRIPESGGDNSVFDIFELTGAAR.pdf]

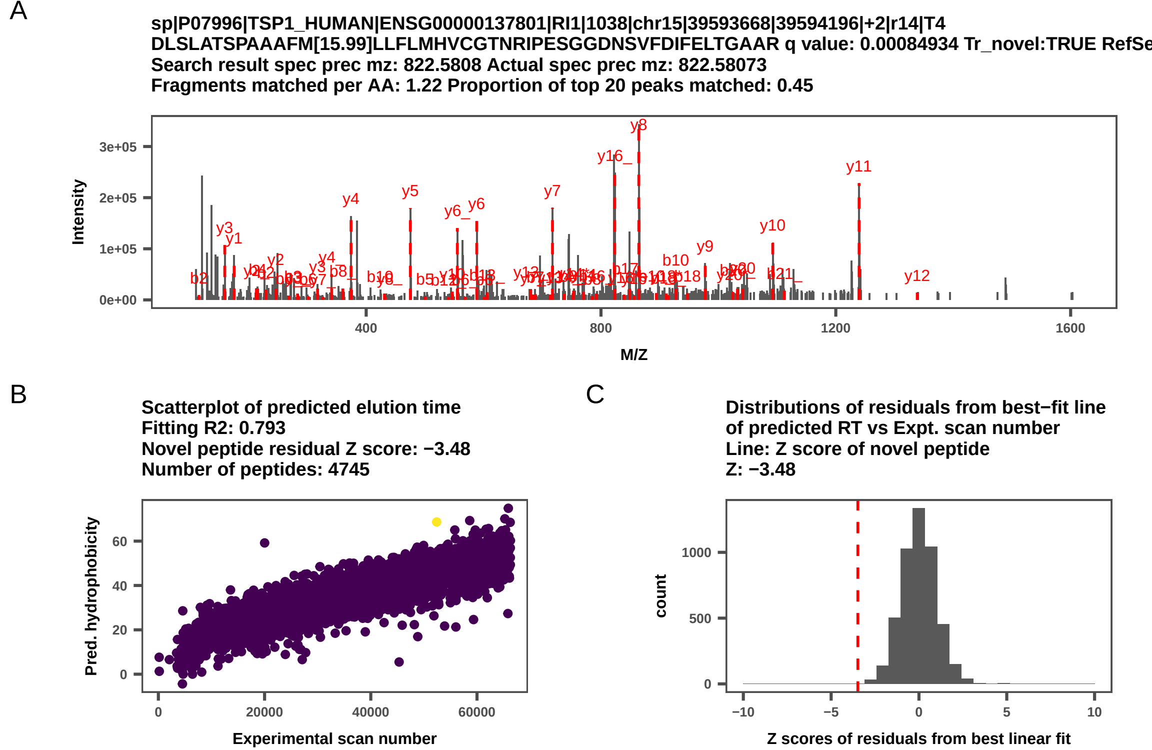

A
sp|P07996|TSP1_HUMAN|ENSG00000137801|RI1|1038|chr15|39593668|39594196|+2|r14|T4
DLSLATSPAAAFM[15.99]LLFLMHVCGTNRIPESGGDNSVFDIFELTGAAR q value: 0.00084934 Tr_novel:TRUE RefSe
Search result spec prec mz: 822.5808 Actual spec prec mz: 822.58073
Fragments matched per AA: 1.22 Proportion of top 20 peaks matched: 0.45
Intensity
3e+05
2e+05
1e+05
0e+00
y8
y16_
y11
y4
y5
y6_
y6
y7
y10
y9
b10
y3
y1
y2
y12
b2
M/Z
400
800
1200
1600
B
Scatterplot of predicted elution time
Fitting R2: 0.793
Novel peptide residual Z score: −3.48
Number of peptides: 4745
Pred. hydrophobicity
60
40
20
0
Experimental scan number
0
20000
40000
60000
C
Distributions of residuals from best−fit line
of predicted RT vs Expt. scan number
Line: Z score of novel peptide
Z: −3.48
count
1000
500
0
Z scores of residuals from best linear fit
−10
−5
0
5
10
